# Cu_1.94_S-Assisted Growth of Wurtzite CuInS_2_ Nanoleaves by In Situ Copper Sulfidation

**DOI:** 10.1186/s11671-015-0996-y

**Published:** 2015-07-15

**Authors:** Chunqi Cai, Lanlan Zhai, Chao Zou, Zhensong Li, Lijie Zhang, Yun Yang, Shaoming Huang

**Affiliations:** Zhejiang Key Laboratory of Carbon Materials, College of Chemistry and Material Engineering, Wenzhou University, Wenzhou, 325027 People’s Republic of China

**Keywords:** CuInS_2_, Wurtzite, Catalyst, Nanoleaves

## Abstract

Wurtzite CuInS_2_ nanoleaves were synthesized by Cu_1.94_S-assisted growth. By observing the evolution of structures and phases during the growth process, Cu_1.94_S nanocrystals were found to be formed after uninterrupted oxidation and sulfidation of copper nanoparticles at the early stage, serving as catalysts to introduce the Cu and In species into CuInS_2_ nanoleaves growth for inherent property of fast ionic conductor. The obtained CuInS_2_ nanoleaves were characterized by scanning transmission electron microscopy, transmission electron microscopy, fast Fourier transform, X-ray diffraction, and energy dispersive X-ray spectroscopy mapping. The enhancement of photoresponsive current of CuInS_2_ nanoleaf film, evaluated by *I*-*V* curves of nanoleaf film, is believed to be attributed to the fast carrier transport benefit from the nature of single crystalline of CuInS_2_ nanoleaves.

**Electronic supplementary material:**

The online version of this article (doi:10.1186/s11671-015-0996-y) contains supplementary material, which is available to authorized users.

## Background

Ternary I-III-VI_2_ groups of compounds are important players in solar energy-harvesting materials [1–3]. Among them, CuInS_2_ is a direct gap semiconductor with a bulk band gap of approximately 1.5 eV and a high extinction coefficient of around 10^5^ cm^−1^ [4, 5]. It is noteworthy that bulk CuInS_2_ at room temperature has the chalcopyrite structure, whereas CuInS_2_ nanocrystals can be additionally synthesized in zincblende and wurtzite structure [6]. Since Pan et al. [7] reported the colloidal synthesis of CuInS_2_ nanocrystals with wurtzite structure via hot injection, numerous research works on the field of metastable wurtzite CuInS_2_ nanocrystals have been reported [4], including the synthesis, phase transformation, and photovoltaic application. Kolny-Olesiak et al. [8] demonstrated the phase transforming from Cu_2_S to wurtzite CuInS_2_ nanocrystals.

The wurtzite CuInS_2_ is constructed as randomly distributed copper and indium over the cation sites of the wurtzite ZnS lattice [6]. The cation disorder allows flexibility of the stoichiometry and a tunable Fermi energy over a wide range, which feature particularly in wurtzite CuInS_2_ nanocrystals for the following device fabrication [9]. While most reports describe the preparation of CuInS_2_ nanocrystals, limited work is available for one-dimensional CuInS_2_ nanomaterials [4, 10]. Semiconductor nanomaterials in one-dimensional morphology provide ideal models to study the relationship between electrical transport, optical, and other properties with dimensionality and size confinement [11–13]. Specifically, one-dimensional nanomaterials could offer continuous charge carrier transport pathways and efficiently promote charge separation, which makes them highly attractive for photocatalytic and photovoltaic applications [14–16]. Thus, one-dimensional nanomaterials comprise an important class of nanomaterials used in electronic and photoelectronic devices, including field-effect transistors, energy harvesting, and sensors [12, 17].

To synthesize one-dimensional nanomaterials in solution, several mechanisms have been developed [11], including catalyst-assisted growth, template-directed growth, and oriented attachment growth. Among them, catalyst-assisted growth [18] exhibited wonderful features to acquire one-dimensional nanomaterials with high crystallinity, tolerating big lattice mismatch between catalysts and targeted nanomaterials. During the growth process, catalyst either formed a liquid eutectic in solution-liquid-solid growth [19], which induces nanowire formation after supersaturation, or enables solid-phase diffusion in supercritical-fluid-liquid-solid growth in which the catalysts remain solid [20]. In these researches, metallic bismuth and indium nanocrystals usually acted as the catalysts [21]. Recently, sulfide Ag_2_Se and Cu_2_S nanocrystals have also been found to be the effective catalysts in the synthesis of one-dimensional nanomaterials for the intrinsic nature of fast ionic conductor [22, 23]. Wang et al. [22] reported Ag_2_Se nanocrystals could be used as catalysts for the growth of semiconductor heterostructures, such as dimeric Ag_2_Se-CdSe and trimeric Ag_2_Se-CdSe-ZnSe. Further, Tang et al. [24] successfully fabricated Cu_2_S-In_2_S_3_ heterostructures by djurleite Cu_1.94_S-assisted growth model, in which the catalyst underwent transformations in crystal structure and composition. Accordingly, Wang et al. [25] proposed the novel solution-solid-solid mechanism for nanowire growth catalyzed by superionic (fast ionic) conductor nanocrystals. By using solution-solid-solid growth, Ag_2_S-CdS, Cu_2_S-ZnS, and Ag_2_Se-ZnSe heterostructures were prepared [26, 27]. In the growth process of one-dimensional nanomaterials, Ag_2_S and Cu_2_S nanocrystals were usually decomposed from single-source molecular precursors and used as catalysts. Then, the target species dissolved into the catalysts and dissolved out after supersaturation. The complicated process in these cases makes one aware that further investigation is needed, for the solubility and fluidity of intermediate species in the catalysts and the supersaturation and condensation of target substances are unique [27, 28]. Thus, there is much room in the exploration of catalysts for the growth of the desired nanomaterials.

Here, we report the catalyst-assisted growth of wurtzite CuInS_2_ nanoleaves in solution by using commercial copper nanoparticles as staring materials. The transformation from copper nanoparticle to copper oxide in oxygen atmosphere underwent quickly at elevated temperature, and then to copper sulfide Cu_1.94_S with the presence of dodecanethiol. Detailed investigation on the growth by monitoring the structures and morphologies of the nanoleaves during the process implied that the formed Cu_1.94_S nanocrystals played the catalytic roles for the CuInS_2_ nanoleaf growth. The structure and composition of CuInS_2_ nanoleaves were also investigated by transmission electron microscopy (TEM), X-ray diffraction (XRD), and energy-dispersive X-ray spectroscopy (EDS). Furthermore, the photoresponsive characteristics of the CuInS_2_ nanoleaf film were also evaluated.

## Methods

## Materials

All chemicals were used as received without further purification. Sodium diethyldithiocarbamate trihydrate (Na(dedc), 99 %), chloroform (99.9 %), and *n*-hexane (95 %) were obtained from J&K, indium nitrate (In(NO_3_)_3_, 99.9 %) from ABCR, oleylamine (OA, C18 content 80–90 %) from Acros, and copper nanoparticle (99.9 %) and 1-dodecanethiol (DT, 98 %) from Alfa.

### Synthesis of In(dedc)_3_ Precursors

In a typical synthesis of In(dedc)_3_, 3 mmol Na(dedc) and 1 mmol In(NO_3_)_3_ were dissolved in 50 mL ionized water, respectively. Then, In(NO_3_)_3_ aqueous solution was mixed with Na(dedc) solution by drop adding, washed three times at least with ionized water and ethanol followed by drying. As-synthesized precursors were stored in desiccator at room temperature.

### Synthesis of CuInS_2_ Nanoleaves

In a typical synthesis of CuInS_2_ nanoleaves, 0.1 mmol (6.4 mg) copper nanoparticles and 0.05 mmol (28.0 mg) In(dedc)_3_ were dispersed in 6.0 mmol (2.0 mL) OA and 16.5 mmol (4.0 mL) DT-loaded flask under magnetic stirring. Then, the flask was vacuumed and filled with oxygen. The procedures were repeated three times and the oxygen flow was maintained during the following reaction. The flask containing the mixture was immersed in an oil bath at 180 °C. The heated solution in the flask showed the color evolution within 1 min, from transparent yellow to light brownish red, implying the formation and decomposition of the Cu-DT complex. After keeping the mixture at the temperature for 60 min, the resulting solution was cooled to room temperature and the samples were washed with *n*-hexane followed by further centrifugation. Aliquots were taken out during the synthesis for monitoring the size and shape evolution of nanoleaves.

### Characterization

The obtained crystalline phases were identified using powder XRD (Bruker, D8 advance, Cu Ka radiation using a curved graphite receiving monochromate), with a step of 0.02° at a speed of 4°/min. The simulated XRD patterns of CuInS_2_ were obtained by using CrystalMaker 2.5.5 programs. Morphology analyses were undertaken using scanning transmission electron microscopy (STEM, FEI Nova NanoSEM 200). TEM, high-angle annular dark-field (HAADF), STEM, and EDS were performed on JEOL 2100F microscope. The samples for TEM, HAADF-STEM, and STEM-EDS were collected by placing a drop of dilute solution of sample in hexane onto carbon-film-supported nickel grids. Composition analysis was performed by EDS (oxford INCA). The two parallel gold electrodes on silicon substrate with quartz layer were used to evaluate electrical property of CuInS_2_ nanoleaves. The interval and length of the two gold electrodes is 5 and 100 μm. Sample was made by drop-casting nanoleaves in chloroform onto the substrate. Annealing process was conducted at 400 °C to remove the attached ligands. The current-voltage characteristics were recorded using a Keithley 4200 Source Meter in the dark and under illumination. The scan voltage was tuned from −10 to 10 V.

## Results and Discussion

**Fig. 1:**
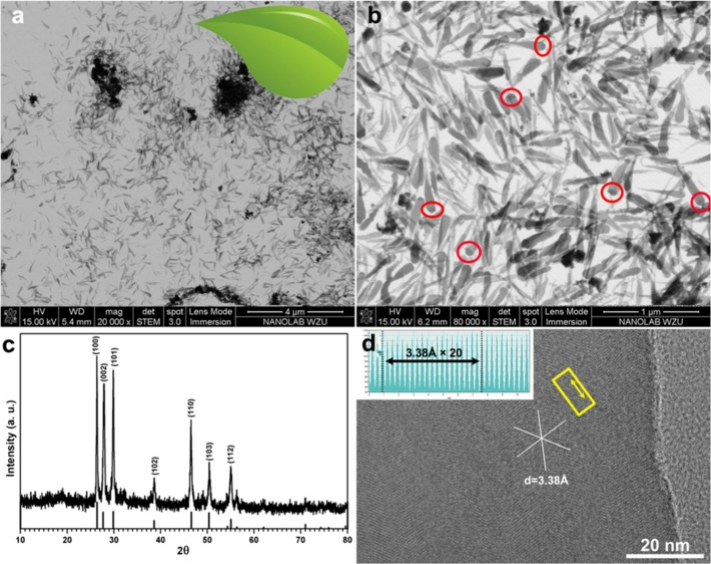
**a**, **b** STEM micrographs of CuInS_2_ nanoleaves. The *red circles* indicate the typical end terminal of the nanoleaves. **c** XRD pattern of as-synthesized CuInS_2_ nanoleaves. The *reference line below* is simulated according to wurtzite CuInS_2_ lattice constants (*a* = 3.9080 Å, *c* = 6.4250 Å). **d** HRTEM micrograph of CuInS_2_ nanoleaves. *Inset* represents line profile integrated along the region enclosed in the *yellow rectangle area*

Typical STEM micrographs of the resultant CuInS_2_ nanomaterials were shown in Fig. 1. The representative micrographs of the as-synthesized samples indicate that the CuInS_2_ nanomaterials have a leaf shape, about 500 nm in length and 100 nm in width. The nanoparticle is observed at the tip of almost every nanoleaf (red circles in Fig. 1b), which is the typical morphology of catalyst-assisted growth. The crystal structure of CuInS_2_ nanoleaves is determined as hexagonal wurtzite phase, originated from wurtzite ZnS by randomly replacing zinc ions with copper or indium ions [7, 8]. The diffraction pattern of wurtzite CuInS_2_ is simulated (*a* = 3.9080 Å, *c* = 6.4250 Å) and listed as reference line in Fig. 1c, for the standard card has not been established. The lattice-resolved high-resolution TEM micrograph (Fig. 1d) shows the single crystal nature of the individual nanoleaves with lattice spacing of 3.38 Å. Accordingly, the (100) plane of the wurtzite CuInS_2_ is resolved. The regularity of lattice spacing in the nanoleaves is also seen from the line profile as inset in Fig. 1d, enclosed from the yellow rectangle area. The distance of 6.76 nm can be represented as 20 × 3.38 Å, which is the lattice spacing of (100) plane in CuInS_2_.

**Fig. 2:**
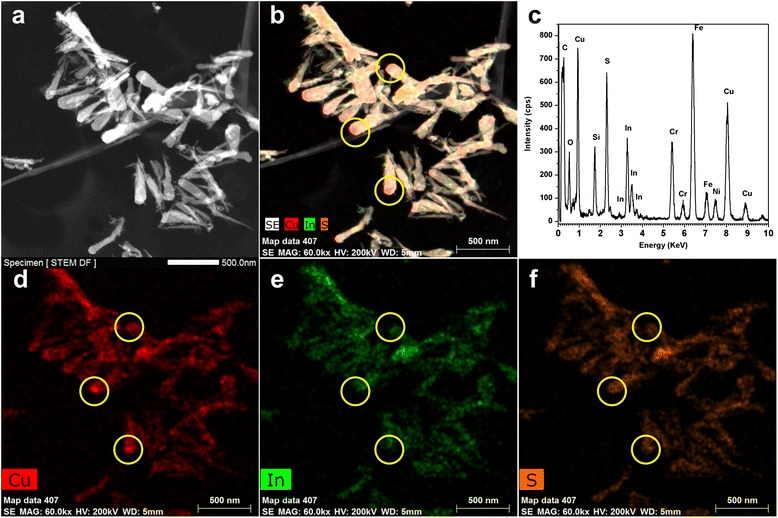
HAADF-STEM micrographs of CuInS_2_ nanoleaves. **a** HAADF micrograph. **b** The composite STEM-EDS micrograph. **c** EDS spectrum collected from CuInS_2_ nanoleaves. Si element peak attributed to EDS detector and Cr, Fe, and Ni element peaks attributed to nonmagnetic nickel grids. **d**–**f** Cu, In, and S of STEM-EDS elemental maps of CuInS_2_ nanoleaves

Figure 2 displays the STEM micrograph and EDS spectrum of CuInS_2_ nanoleaves. The composition of the nanoleaves was mainly identified as Cu, In, and S elements. Chemical analyses with nanoscale spatial resolution are performed to clarify the sample compositions and elemental distributions in individual. The 2D-projected elemental maps for three atoms, shown in Fig. 2d–f and Additional file 1: Fig. S1, demonstrate the reasonable separated distribution of three atoms among the nanoleaves. Indicated by yellow circles in Fig. 2b, d–f and Additional file 1: Fig. S1, enhanced copper content in head part of nanoleaves is thought to be attributed to copper sulfide catalyst and will be discussed later. The composition of the head part in the nanoleaf was identified as Cu and S elements with ratio of 61/39. Meanwhile, element ratio 26/29/45 of Cu/In/S was identified in the body part of nanoleaf. Also, line scan across the single CuInS_2_ nanoleaf testified the difference between head and body parts (Additional file 1: Fig. S2 and S3). Copper content has considerable high ratio in the head part than body part, while indium content could be negligible in the head part compared with that in the body part.

**Fig. 3:**
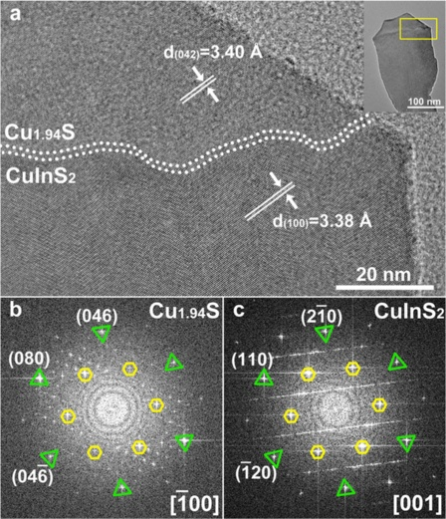
Interface of Cu_1.94_S catalyst and wurtzite CuInS_2_ nanoleaves. **a** Lattice-resolved HRTEM micrograph, *inset* shows the single banana leaf. **b**, **c** The corresponding fast Fourier transform (FFT) patterns of Cu_1.94_S and CuInS_2_ parts in nanoleaf, respectively

A typical HRTEM micrograph of the tip in CuInS_2_ nanoleaf is shown in Fig. 3a. The interface between head and body parts in the nanoleaf, indicated by the parallel dotted lines, is clearly revealed. The two FFT patterns corresponding to head and body parts are noticeably dissimilar and are indexed to monoclinic Cu_1.94_S and wurtzite CuInS_2_, respectively. Lattice spacing with 3.40 Å in the head and 3.38 Å in the body is corresponded to the (042) plane of Cu_1.94_S crystal and (100) plane of CuInS_2_ crystal, respectively. An interplanar distance analysis based on the HRTEM and FFT pattern shown in Fig. 3 suggests that the (201̄0) plane of CuInS_2_ nanoleaf epitaxially attaches to the (046) plane of the Cu_1.94_S head, which demonstrates the grain boundary is composed of (21̄0) planes of CuInS_2_ and (046) planes of Cu_1.94_S. Based on above analysis, an atomic packing model that depicts the epitaxial attachment of the CuInS_2_ body to Cu_1.94_S head is proposed. The corresponding interface orientation relationships between the CuInS_2_ and Cu_1.94_S are (010)_CuInS2_||(042̄)_Cu1.94S_ and [001]_CuInS2_|| [1̄00]_Cu1.94S_, respectively. The values of lattice mismatch are [(3.38–3.40)/3.40 × 100 %] = −0.59 % for (010)_CuInS2_||(042̄)_Cu1.94S_ direction, and [(3.38–3.36)/3.38 × 100 %] = 0.59 % for (001)_CuInS2_||(8̄00)_Cu1.94S_ direction, respectively. The relatively small lattice mismatch along both directions enables epitaxial growth of CuInS_2_ nanoleaf on Cu_1.94_S head.

**Fig. 4:**
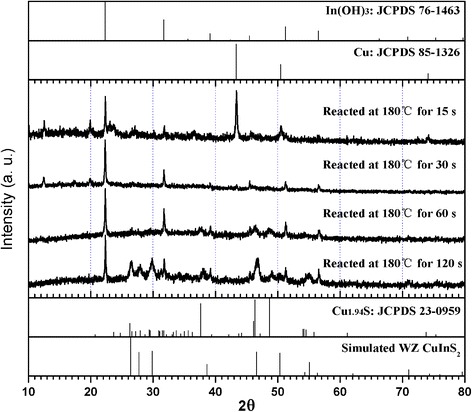
XRD patterns of samples at different reaction stages. The upper and lower reference lines are In(OH)_3_, Cu, Cu_1.94_S, and simulated wurtzite CuInS_2_, respectively

**Fig. 5:**
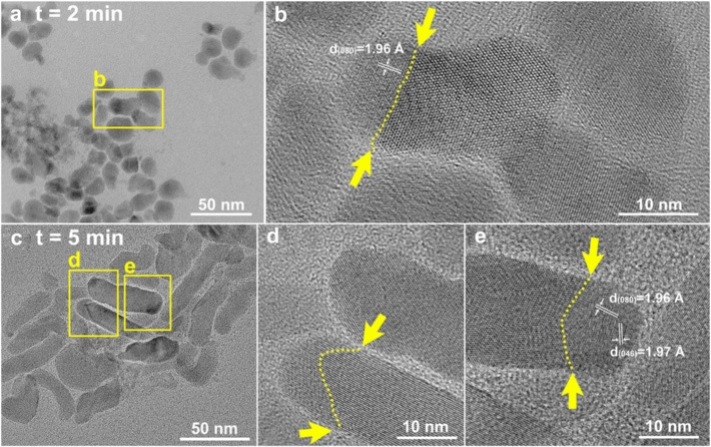
Growth process of CuInS_2_ nanoleaves. **a**, **b** HRTEM micrographs, *t* = 2 min. **c**–**e** HRTEM micrographs, *t* = 5 min. The *yellow arrows* and *dotted lines* indicate the interfaces between head and body parts of the CuInS_2_ nanomaterials

To explore the growth of CuInS_2_ nanoleaves, the phase and morphological evolution of samples at different reaction stages was investigated by XRD and TEM (Figs. 4 and 5). Metal copper and indium hydroxide were the main phase of the solid product within 15 s after the reaction system immersed into the oil bath at 180 °C, and the latter was originated from the reaction between oleylamine and indium precursors. The signal of djurleite Cu_1.94_S emerged as the reaction lasted for 60 s. The coexistence of wurtzite CuInS_2_, indium hydroxide, and djurleite Cu_1.94_S was observed in the product after 120 s. It is supposed that djurleite Cu_1.94_S catalyzed the growth for the nanoleaves. By contrast, copper phase kept unchanged at the early stage under nitrogen atmosphere (the absence of oxygen) in the controlled experiment. The transformation from copper nanoparticle to copper oxide in oxygen atmosphere underwent at elevating temperature process, and then to Cu_1.94_S rapidly after the reaction with dodecanethiol. It is thought that sulfidation has higher reaction rate than oxidation, though the latter possesses the priority, which also explains the fail trial on the confirmation of copper oxide by XRD analysis. In vapor system, the role of oxygen pressure during the transformation from metal copper to copper sulfide has been clarified [29, 30]. Monoclinic Cu_2_S nanowire arrays were grown on Cu foil substrates with the aid of H_2_S gas. In consideration that catalytic roles played by Cu_1.94_S were validated only if wurtzite CuInS_2_ and djurleite Cu_1.94_S coexist, the sample at 2 min was chose as the target. As shown in Fig. 5a, the samples at the early stage appear as acorns. Lattice-resolved HRTEM micrographs (Fig. 5b) clearly reveal the interface between the head and body parts of samples, indicated by the yellow dotted lines. With the growth proceeding (Fig. 5c–e), the body parts of CuInS_2_ samples grew with the gradual increase of length, whereas the Cu_1.94_S head parts and the interfaces kept.

The Cu_1.94_S head part in CuInS_2_ nanomaterial is deduced to be catalyst, which is the typical role in the synthesis of one-dimensional nanomaterial by the mechanisms of solution-liquid-solid and vapor-liquid-solid growth [19, 31]. Recently, superionic conductor nanocrystals, such as Ag_2_S, Ag_2_Se, and Cu_2_S, are found to be efficient catalysts in the growth of nanowires and heteronanostructures for their intrinsic nature [22, 25–27, 32]. Also, new mechanism has been proposed as solution-solid-solid mode [25]. The superionic conductor catalysts have enough cation vacancies in their lattice with high cation mobility in the rigid anionic sublattice. It has been demonstrated that djurleite Cu_1.94_S nanocrystal can catalyze the growth of Cu_2_S-In_2_S_3_, Cu_1.94_S-CdS, and Cu_2_S-PbS heterostructures for the intrinsic cationic deficiencies [23, 24, 33, 34]. In the present work, the catalyst Cu_1.94_S nanocrystal introduces Cu(I) and In(III) species into the vacant sites of the crystal lattice, then condenses and crystallizes successively after saturation from the favorable facet of the catalyst to minimize the interfacial energy. As calculated from the proposed atomic packing model, lattice mismatches are as small as 0.59 %. We can deduce that grain boundary with low interfacial energy between djurleite Cu_1.94_S head and wurtzite CuInS_2_ body is formed.

Comparatively, size enlargement of Cu_1.94_S, from 10 nm in width at 2 min (Fig. 5a, b) to 100 nm at 60 min (Fig. 3a), provided further evidence for catalyst-assisted growth of CuInS_2_ nanoleaves. If seed-mediated growth model is employed in the present system, the size of Cu_1.94_S should be stable as the targeted material only grows epitaxially on the specific facet of Cu_1.94_S seed. From this point, the growth of the targeted materials just involves the first atomic layer epitaxial growth on seed, then transforms into conventional crystal growth. Thus, the subsequent growth of CuInS_2_ nanoleaves by seed-mediated growth makes no difference to the seed, either in composition or in size.

**Fig. 6:**
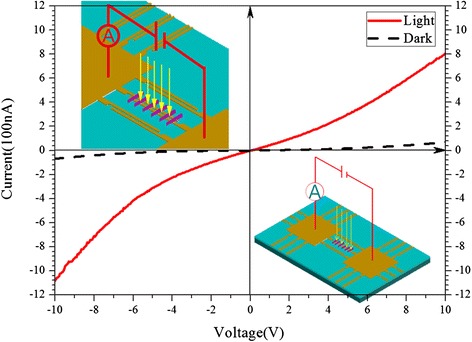
*I*-*V* characteristic of drop-casting thin film of CuInS_2_ nanoleaves in the dark (*dark*) and under illumination by a lamp (*red*). *Insets* show schematic illustration of nanoleaf-based device

To further evaluate the optoelectronic properties of the CuInS_2_ nanoleaves, thin films by drop-casting nanoleaves solutions on inter-digitated electrode (IDE) silicon substrate test chips were fabricated [35]. After that, annealing process was conducted at 400 °C to remove the attached ligands, OA, and DT, facilitating the carrier transportation among nanoleaves. Shown in Fig. 6, the *I*-*V* curve demonstrates the photoresponsive property of thin films composed of CuInS_2_ nanoleaves. The drop-casting CuInS_2_ film exhibits observable photoresponsive property, 11-fold increase from 7.2 × 10^−8^ A at 10 V in the dark to 8.1 × 10^−7^ A under illumination. The enhancement of photoresponsive current of CuInS_2_ films in this work is deduced to be beneficial from fast carriers transport for their nature of single crystalline. Tang and Sargent [35] reported that colloidal CuInSe_2_, CuGaSe_2_, and Cu(InGa)Se_2_ nanoparticle-based thick films showed photoresponse, from 2–10-fold current increase compared dark with lamp. And also, ZnO nanocrystal-based field-effect transistors exhibited much improved semiconducting properties with spin-coated ZnO thin-films by tuning the shape from zero-dimensional nanocrystals to one-dimensional nanorods [36]. In contrast to nanocrystals, nanoleaves with one-dimensional morphology do not have grain boundary along their length [12, 36, 37], which might have faster carrier transport than percolation through a random polycrystalline network in nanocrystals, and thus enhancing photoresponse.

## Conclusions

We demonstrated the catalyst-assisted growth of wurtzite CuInS_2_ nanoleaves in solution by using commercial copper nanoparticles as staring materials. The transformation from copper nanoparticle to copper oxide and then copper sulfide Cu_1.94_S underwent quickly in the presence of oxygen atmosphere at the elevated temperature. Then, Cu_1.94_S nanocrystals played the catalytic roles for the growth of wurtzite CuInS_2_ nanoleaves. The 2D-projected elemental maps for three elements demonstrated the evenly distribution of those elements among CuInS_2_ nanoleaves. Photoresponses of CuInS_2_ nanoleaves were evaluated by *I*-*V* measurements, 11-fold increase compared with that in the dark. The enhancement of photoresponsive current of CuInS_2_ film is believed to be attributed to one-dimensional single crystalline nature of CuInS_2_ nanoleaves.

## Additional file

Additional file 1: Figure S1. HAADF-STEM images of CuInS_2_ nanoleaf. (a) the composite STEM-EDS micrograph, (b) EDS spectra collected from head and body parts of nanoleaf. Mo and Si element peaks attributed to molybdenum grid and EDS detector. (c-e) STEM-EDS elemental maps of Cu, In, and S, respectively. The yellow cycles indicates the head part of nanoleaf. **Figure S2.** HAADF-STEM image of CuInS_2_ nanoleaf. (a) SE image, (b) EDS line scan profile. The analysis was made from the head part toward the body part of nanoleaf, as indicated by hollow blue arrow. **Figure S3.** HAADF-STEM image of CuInS_2_ nanoleaf. (a) SE image, (b) EDS line scan profile. The analysis was made from the head part toward the body part of nanoleaf, as indicated by hollow blue arrow.
