# Supplementary material for: Cu1.94S-Assisted Growth of Wurtzite CuInS2 Nanoleaves by In Situ Copper Sulfidation
Source: Nanoscale Res Lett. 2015 Jul 15;10:294. doi: 10.1186/s11671-015-0996-y (PMC4516149; doi:10.1186/s11671-015-0996-y)
Supplement: Additional file 1: Figure S1. — HAADF-STEM images of CuInS2 nanoleaf. (a) the composite STEM-EDS micrograph, (b) EDS spectra collected from head and body parts of nanoleaf. Mo and Si element peaks attributed to molybdenum grid and EDS detector. (c-e) STEM-EDS elemental maps of Cu, In, and S, respectively. The yellow cycles indicates the head part of nanoleaf. Figure S2. HAADF-STEM image of CuInS2 nanoleaf. (a) SE image, (b) EDS line scan profile. The analysis was made from the head part toward the body part of nanoleaf, as indicated by hollow blue arrow. Figure S3. HAADF-STEM image of CuInS2 nanoleaf. (a) SE image, (b) EDS line scan profile. The analysis was made from the head part toward the body part of nanoleaf, as indicated by hollow blue arrow. [file 11671_2015_996_MOESM1_ESM.docx]

Cu_1.94_S-assisted growth of wurtzite CuInS_2_ nanoleaves by *in situ* copper sulfidation

Chunqi Cai, Lanlan Zhai, Chao Zou*, Zhensong Li, Lijie Zhang, Yun Yang and Shaoming Huang*

Zhejiang Key Laboratory of Carbon Materials, College of Chemistry and Material Engineering, Wenzhou University, Wenzhou 325027, P. R. China.

Fax: 86 577 8837 3064; Tel: 86 577 8837 3064;

E-mail: zouchao@wzu.edu.cn, smhuang@wzu.edu.cn


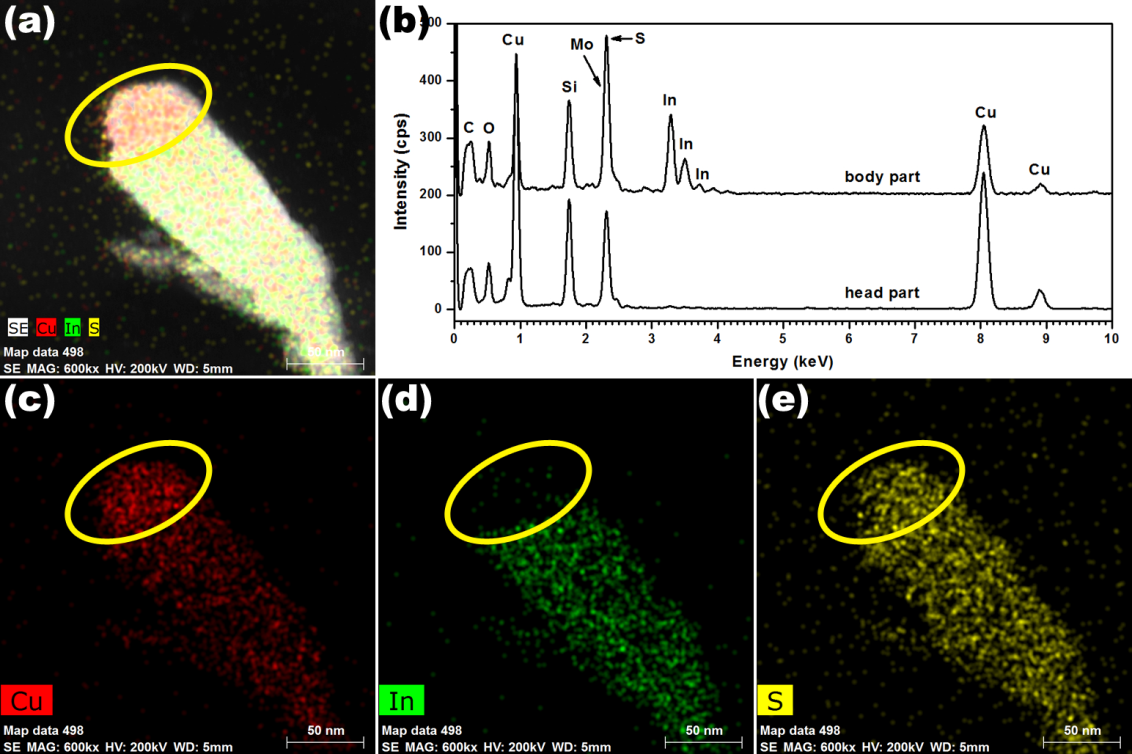


Figure S1. HAADF-STEM images of CuInS_2_ nanoleaf. (a) the composite STEM-EDS micrograph, (b) EDS spectra collected from head and body parts of nanoleaf. Mo and Si element peaks attributed to molybdenum grid and EDS detector. (c-e) STEM-EDS elemental maps of Cu, In, and S, respectively. The yellow cycles indicates the head part of nanoleaf.


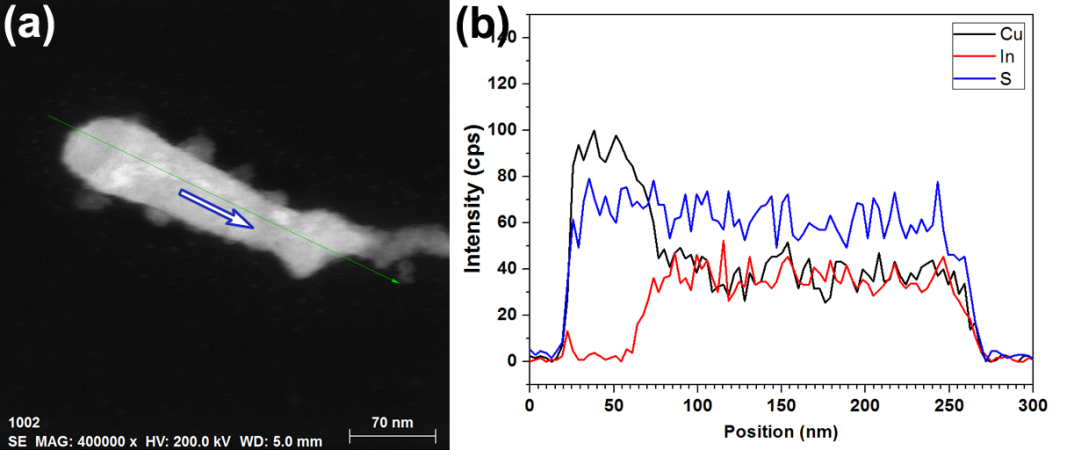


Figure S2. HAADF-STEM image of CuInS_2_ nanoleaf. (a) SE image, (b) EDS line scan profile. The analysis was made from the head part toward the body part of nanoleaf, as indicated by hollow blue arrow.


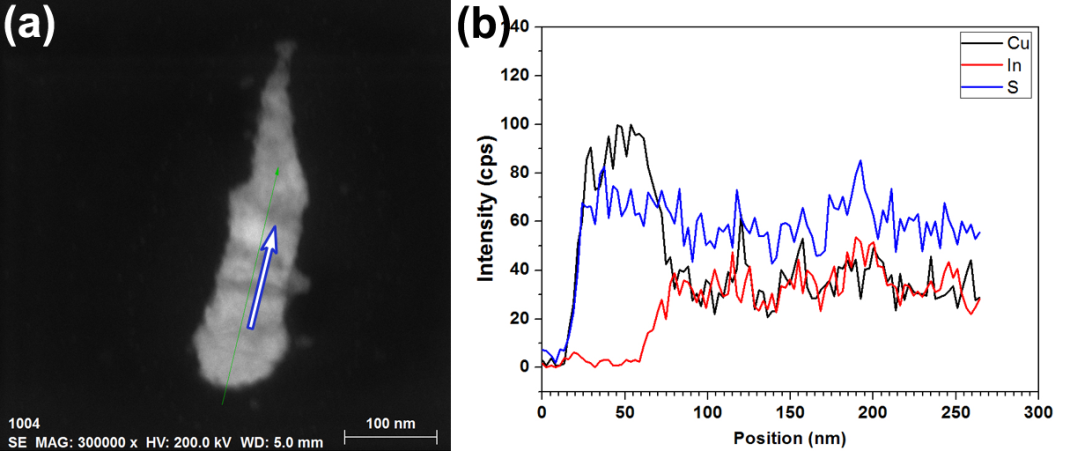


Figure S3. HAADF-STEM image of CuInS_2_ nanoleaf. (a) SE image, (b) EDS line scan profile. The analysis was made from the head part toward the body part of nanoleaf, as indicated by hollow blue arrow.
